# Structural, Optical, Electrical and Photocatalytic Investigation of n-Type Zn^2+^-Doped α-Bi_2_O_3_ Nanoparticles for Optoelectronics Applications

**DOI:** 10.1021/acsomega.3c10521

**Published:** 2024-05-17

**Authors:** Asad ur Rehman Khan, Muhammad Ramzan, Seham J. F. Alanazi, Amal M. Al-Mohaimeed, Shahzaib Ali, Muhammad Imran, Muhammad Abdul Majid, Muhammad Hassan Sarfraz

**Affiliations:** † Institute of Physics, Baghdad ul Jadeed Campus, The Islamia University of Bahawalpur, Bahawalpur 63100, Pakistan; ‡ Department of Chemistry, College of Science, King Saud University, P.O. Box 22452, Riyadh 11495, Saudi Arabia; § Department of Physics, Quaid-i-Azam University, Islamabad 45320, Pakistan; ∥ Department of Electronics, Government College University Lahore, Lahore 54000, Pakistan; ⊥ Botnar Institute of Musculoskeletal Sciences, Nuffield Department of Orthopaedics, Rheumatology and Musculoskeletal Sciences, University of Oxford, Oxford OX3 7LD, U.K.

## Abstract

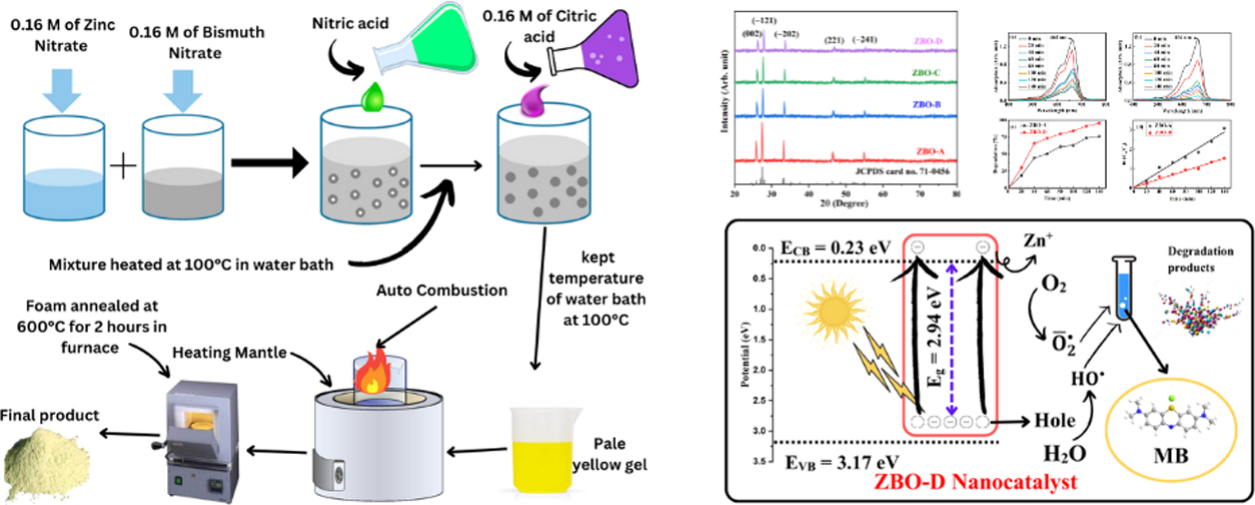

Herein, n-type pure and Zn^2+^-doped monoclinic bismuth oxide nanoparticles were synthesized by the citrate sol–gel method. X-ray diffraction (XRD), scanning electron microscopy (SEM), Fourier transform infrared spectroscopy (FTIR), photoluminescence (PL) analysis, ultraviolet–visible (UV–vis) spectroscopy, and Hall effect measurements were used to study the effect of Zn^2+^ on the structural, optical, and electrical properties of nanoparticles. XRD revealed the monoclinic stable phase (α-Bi_2_O_3_) of all synthesized samples and the crystallite size of nanoparticles increased with increasing concentration of dopant. Optical analysis illustrated the red shift of absorption edge and blue shift of band gap with increasing concentration of dopant. Hall Effect measurements showed improved values (2.79 × 10^–5^ S cm^–1^ and 6.89 cm^2^/V·s) of conductivity and mobility, respectively, for Zn^2+^-doped α-Bi_2_O_3_ nanoparticles. The tuned optical band gap and improved electrical properties make Zn^2+^-doped α-Bi_2_O_3_ nanostructures promising candidates for optoelectronic devices. The degradation of methylene blue (MB, organic dye) in pure and zinc-doped α-Bi_2_O_3_ was investigated under solar irradiation. The optimum doping level of zinc (4.5% Zn^2+^-doped α-Bi_2_O_3_) reveals the attractive photocatalytic activity of α-Bi_2_O_3_ nanostructures due to electron trapping and detrapping for solar cells.

## 1 Introduction

Since a few decades, scientific researchers have shown significant interest in metal oxides due to their environmentally friendly behavior, thermal stability, ferroelectric properties, mechanical strength, and biocompatibility. These metal oxide nanostructures have significant importance in a number of applications such as in nanomedicines, electrochemical sensors, supercapacitors, fluoresce, optoelectronics, and much more.^1−10^

Bismuth chalcogenides (Bi_2_E_3_, E = Te, O, Se) are compounds that have noteworthy and attractive features based on electrical and optical properties. Bi_2_E_3_ compounds are semiconductors with novel properties, which are used in different industrial applications. Among the Bi_2_E_3_ family, bismuth oxide (Bi_2_O_3_) is of great interest because it has six crystallographic polymorphs: α-Bi_2_O_3_ (monoclinic phase), δ-Bi_2_O_3_ (face-centered cubic phase), γ-Bi_2_O_3_ (body-centered cubic phase), ω-Bi_2_O_3_ (triclinic phase), ε-Bi_2_O_3_ (orthorhombic phase), and β-Bi_2_O_3_ (tetragonal phase).^11,12^ Owing to its excellent environmental stability, high carrier mobility, and appropriate band gap, Bi_2_O_3_ is a promising candidate for optoelectronic and electronic devices.^9^ Bi_2_O_3_ is a semiconductor material with a wide band gap in the range between 2 and 3.96 eV.^13^ Optical band gap and electrical conductivity are the most important features, while the optoelectronics applications are under investigation. Bi_2_O_3_ is also known as an amphoteric semiconductor because it can show both p-type and n-type conductivity, which depends upon the method of preparation. The synthesis methods of Bi_2_O_3_ and noble metal oxide nanostructures are similar, in which decomposition of precursors occurs to yield the nuclei.^14^ Bi_2_O_3_ nanostructures can be prepared by coprecipitation,^15^ solution combustion,^16^ hydrothermal, probe sonication,^17^ and sol–gel techniques.^18^ Among all mentioned techniques, the sol–gel method is known to be an inexpensive, ingenious, robust, and momentous method, by which size-controlled nanostructures can be prepared within a short time. Doping of metal oxides opened new windows for multifarious applications in optoelectronic devices, sensors, and displays.^19−21^ Doping of HO^3+^, Tb^3+^, and Sm^3+^ in Bi_2_O_3_ was investigated by Vishwakarma, et al.,^22^ Dixit, et al.,^23^ and Ashwini, et al.,^16^ respectively.

A number of research groups have investigated the effect of transition metal doping in Bi_2_O_3_ nanostructures, but very few have explained the effect of metal doping in Bi_2_O_3_. In the present research work, n-type α-Bi_2_O_3_ nanoparticles doped with different volume percentages (0, 1.5, 3.5, and 5.5) of zinc (Zn^2+^ ions) were synthesized via the citrate sol–gel method. The structural, optical, and electrical properties of the synthesized zinc-doped α-Bi_2_O_3_ nanoparticles were investigated for optoelectronics applications. The photocatalytic analysis of pure and Zn^2+^-doped α-Bi_2_O_3_ nanoparticles was carried out by degradation of the pollutant (MB) in solution under solar illumination.

## 2 Materials and Methods

### 2.1 Synthesis of Pure and Zn-Doped Bi_2_O_3_ Nanoparticles

**Figure 1:**
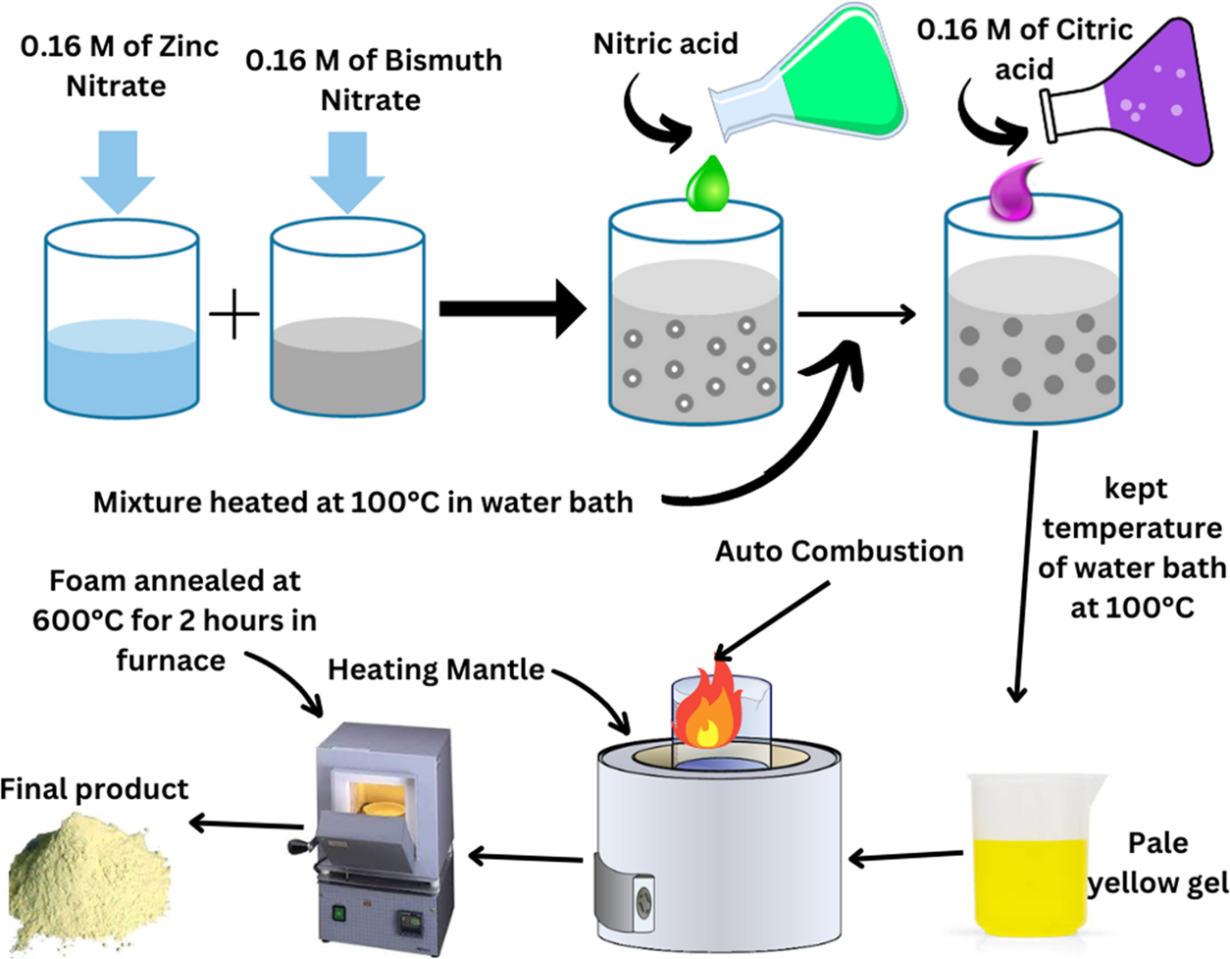
Schematic representation of Zn-doped Bi_2_O_3_ nanoparticles’ synthesis.

All of the chemical reagents were purchased from Sigma-Aldrich and used without any further purification. Zn_*x*_Bi_2–*x*_O_3_, for pure Bi_2_O_3_ (*x* = 0.00) and for zinc-doped Bi_2_O_3_ with doping of Zn taken in different percentages such as *x* = 0.015, 0.03, and 0.045, nanoparticles were successfully developed by the citrate sol–gel method. High-purity bismuth nitrate pentahydrate (Bi(NO_3_)_3_·5H_2_O, ≥98%) and zinc nitrate hexahydrate (Zn(NO_3_)_2_·6H_2_O, ≥99%) were used as initial precursors for Bi and Zn, respectively. In the first step, pure Bi_2_O_3_ nanoparticles were fabricated. A 0.16 M solution of Bi(NO_3_)_3_·5H_2_O was prepared in nitric acid (HNO_3_), and further, this solution was diluted in HNO_3_ in the ratio 1:3. The obtained mixture was heated at 100 °C in a water bath. 0.16 M (equimolar) citric acid (C_6_H_8_O_7_) was added to the hot mixture, and the temperature of the water bath was kept at 100 °C until a gel of pale yellow color formed. A heating mantle was used to heat the formed gel and autocombustion took place to convert the gel into foam to obtain the final product. The final products were annealed at 600 °C for 2 h in a muffle furnace to obtain the improved phase ordering of metal oxides. In the second step, Zn-doped Bi_2_O_3_ was fabricated with the same process. A 0.16 M solution of Zn(NO_3_)_2_·6H_2_O in HNO_3_ was mixed with a 0.16 M solution of Bi(NO_3_)_3_·5H_2_O in HNO_3_ in different volume percentages (1.5, 3, and 4.5) by keeping the volume constant, before diluting in HNO_3_ in the ratio 1:3. The samples were denoted as ZBO-A, ZBO-B, ZBO-C, and ZBO–D for pure Bi_2_O_3_, 1.5, 3, and 4.5 volume percentage doping of Zn in Bi_2_O_3_ nanoparticles, respectively. Figure 1 shows the schematic representation of the synthetic methodology for Zn-doped Bi_2_O_3_ nanoparticles.

### 2.2 Characterization Tools

Structural analysis of fabricated nanoparticles was done using Bruker d8 (Cu–Kα, 1.54 Å) X-ray diffraction (XRD). The morphology and elemental composition of nanoparticles were collected by a scanning electron microscope (SEM), Hitachi SU-70. Optical investigation was carried out using a ultraviolet–visible (UV–vis) dual beam spectrophotometer, Lambda 25, PerkinElmer. Fourier transform infrared (FTIR) spectroscopy was carried out using PerkinElmer spectrum 2. A Horiba RAM-HR800 microscope fitted with a HE-Cd UV laser (29 mW power, 400 nm) was used to perform photoluminescence (PL) measurements. Electrical analysis was carried out using the NANO–CHIP Reliabilty grade Hall effect system; the nanoparticles were coated on glass substrates of 1 cm^2^ area and contact was made by indium metal at four corners.

### 2.3 Photocatalytic Analysis

To investigate the photocatalytic activity of bismuth oxides, 0.2 g of two selected samples ZBO-A (pure α-Bi_2_O_3_) and ZBO–D (4.5% doped Zn^2+^, α-Bi_2_O_3_) were mixed in 100 mL of 1 mg/1 MB and added in a quartz photoreactor. The prepared aqueous solution was mixed for 2 h in darkness and then irradiated under sunlight with 950 ± 25 Wm^–2^ fluctuations. To check the level of MB, samples were collected after every 20 min and centrifuged to separate the unmixed nanoparticles. The remaining solution in the centrifuge of MB used to measure the optical absorbance at 464 nm. The degradation (%) of the dye (MB) in the absence and presence of the catalysts was calculated by the following equation.^24^
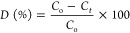
1

*C*_o_ and *C_t_* are the concentrations of the dye (MB) before and after the illumination under sunlight, respectively.

## 3 Results and Discussion

### 3.1 XRD Analysis

**Figure 2:**
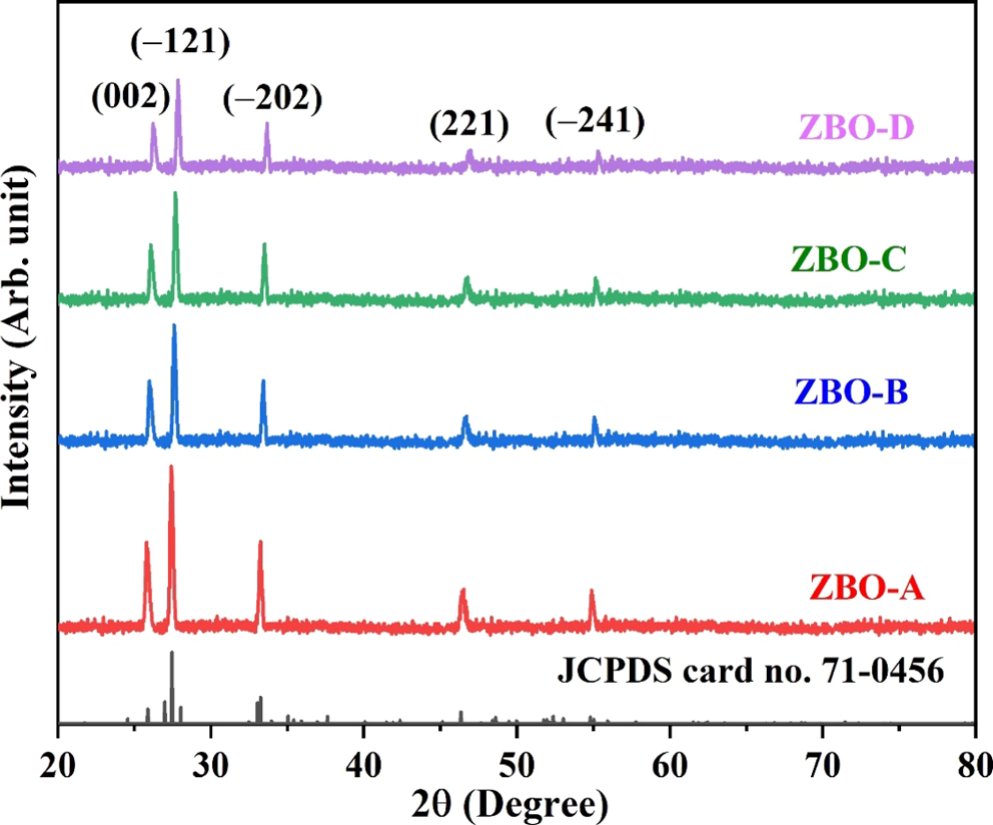
XRD spectra of pure α-Bi_2_O_3_ and Zn^2+^-doped α-Bi_2_O_3_ nanoparticles.

The crystal structure of pure bismuth oxide (ZBO-A) and Zn-doped bismuth oxide (ZBO-B, ZBO-C, and ZBO–D) nanoparticles studied by XRD spectra and XRD patterns of the fabricated samples are shown in Figure 2. The diffraction peaks (002), (−121), (−202), (221), and (−241) illustrated that the synthesized nanoparticles are of monoclinic structure (α-Bi_2_O_3_) and correspond to JCPDS Card No. 71–0465. The diffraction peaks related to Zn and its oxides are not observed in the XRD spectrum, which illustrates that the monoclinic structure of α-Bi_2_O_3_ was not altered by the doping of Zn.^18^ The crystallite size “*D*” of the fabricated samples was calculated using Scherer’s formula^25^eq 2 for the diffraction peak (−121). The lattice parameters “*a*”, “*b*”, and “*c*” were also calculated using the following eq 2.^26^
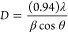
2where “λ” is the wavelength (Cu–Kα radiation 1.54 Å), “θ” is the angle of diffraction, and “β” represents the full width at half-maximum (radians).
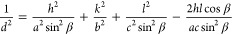
3

**Figure 3:**
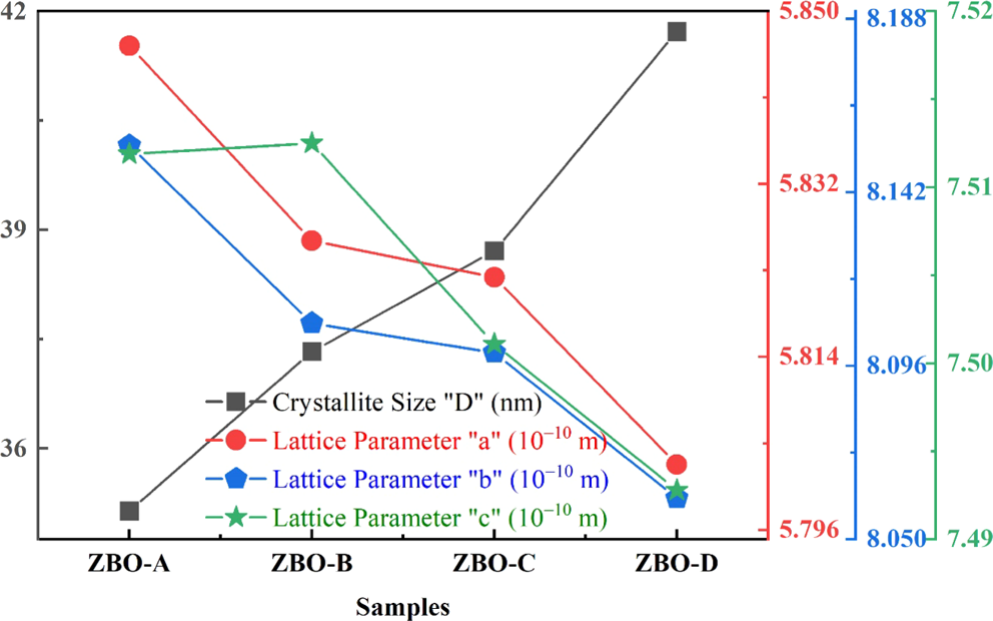
Crystallite size “*D*” and lattice parameters of pure and Zn^2+^-doped α-Bi_2_O_3_ nanoparticles.

The crystallite size of pure α-Bi_2_O_3_ nanoparticles increases from 35.14 to 41.72 nm with increasing volume concentration of Zn, as shown in Figure 3. The increase in crystallite size due to Zn doping because of the Zn^2+^ ion of 0.075 nm ionic radius caused the foreign contaminants’ distortion in the host α-Bi_2_O_3_ lattice at the place of the Bi^3+^ ion of 0.1034 nm ionic radius.^27^ Moreover, lattice parameters “*a*”, “*b*”, and “*c*”, unit cell volume, and β angle were also calculated and the calculated values are shown in Table 1. Unit cell volume and lattice parameters did not exhibit the monotonic variation upon increasing the concentration of Zn. The lattice parameters are also shown in Figure 3. Substitutional doping of Zn^2+^ ions is dominant in the α-Bi_2_O_3_ crystal because the unit cell volume decreases with increasing concentration of Zn. If the interstitial doping of Zn^2+^ ions is dominant, then expansion of unit cell volume occurs,^28^ but in the present study, the substitutional doping is dominant.

**Table 1:** XRD Parameters of the Synthesized Nanoparticles

samples ID	ZBO-A	ZBO-B	ZBO-C	ZBO–D
crystallite size “*D*” (nm)	35.14	37.33	38.71	41.72
lattice parameter “*a*” (Å)	5.8464	5.8261	5.8223	5.8028
lattice parameter “*b*” (Å)	8.1544	8.1073	8.0995	8.0609
lattice parameter “*c*” (Å)	7.5119	7.5125	7.5011	7.4958
unit cell volume (Å^3^)	329.65	326.69	325.73	322.9
β angle (deg)	113	112.97	112.95	112.93

### 3.2 FTIR Analysis

**Figure 4:**
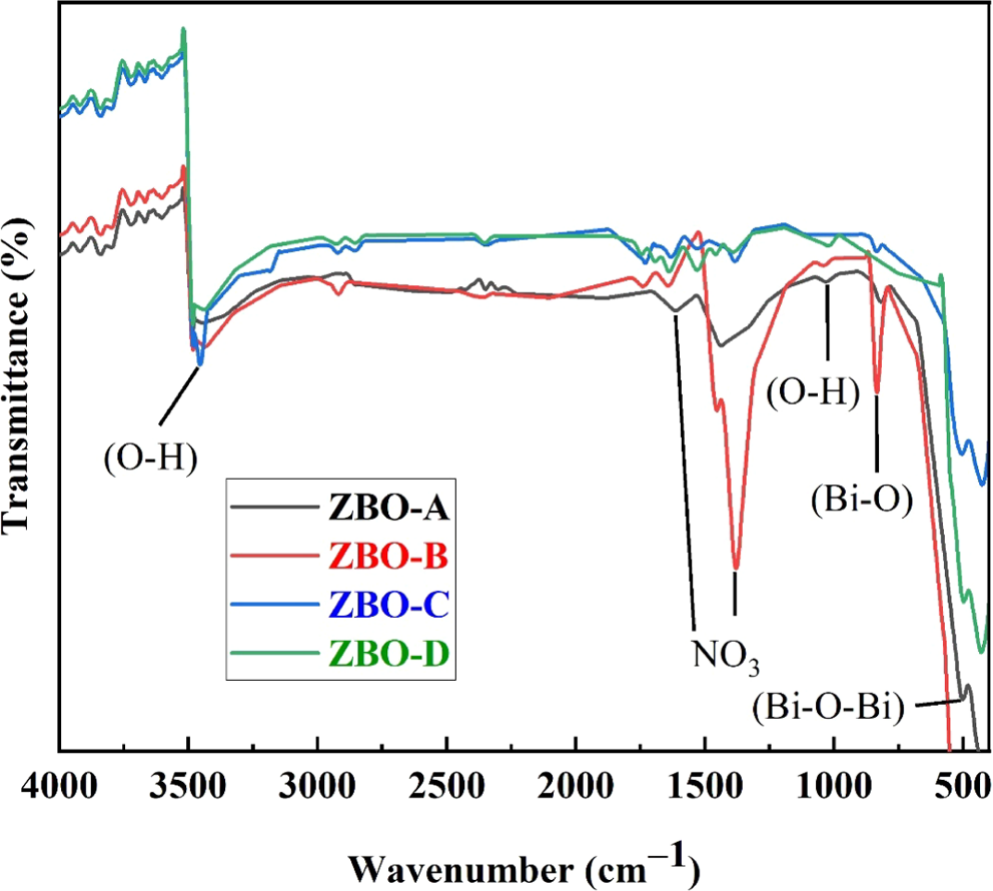
FTIR spectra of pure and Zn^2+^-doped α-Bi_2_O_3_ nanoparticles.

FTIR spectroscopy is a nondestructive technique used to investigate the functional groups in the subjected samples. Figure 4 exhibits the FTIR spectra of pure and Zn^2+^-doped α-Bi_2_O_3_ nanoparticles. All of the synthesized nanoparticles exhibited the absorption bands of OH (hydrogen-bonded) stretching at 3430 cm^–1^.^29,30^ The absorption bands at 3430 cm^–1^ were attributed to the OH group in the synthesized samples due to deoxygenation. The vibration modes appearing at 12,000–1700 cm^–1^ represented the interlayer nitrate (NO_3_ group). The stretching vibration of normal OH was also observed at 1080 cm^–1^, which is due to the presence of water in the Bi–O lattice.^31,32^ Absorption bands due to interatomic vibrations are produced mostly below 1000 cm^–1^ in metal oxides. Stretching vibrations of Bi–O bonds were observed at 840 cm^–1^ in pure and Zn^2+^-doped α-Bi_2_O_3_ nanoparticles. Meanwhile, the peaks originated in 400–700 cm^–1^ are associated with metal oxygen vibrations (Bi–O–Bi). In Figure 4, it is shown that the intensity of Bi–O bond stretching vibrations significantly increased with increasing concentration of Zn^2+^. This progressive enhancement is owing to the variation of the defect state density around the Bi ions when Zn^2+^ ions are doped in the Bi–O lattice. The peaks of Bi–O stretching vibrations illustrated that the desired nanostructures were developed successfully and these spectral peaks are attributed to specific bonding modes or molecular vibrations in nanostructures, which are essential for investigating their potential applications.^33^

### 3.3 Raman Analysis

**Figure 5:**
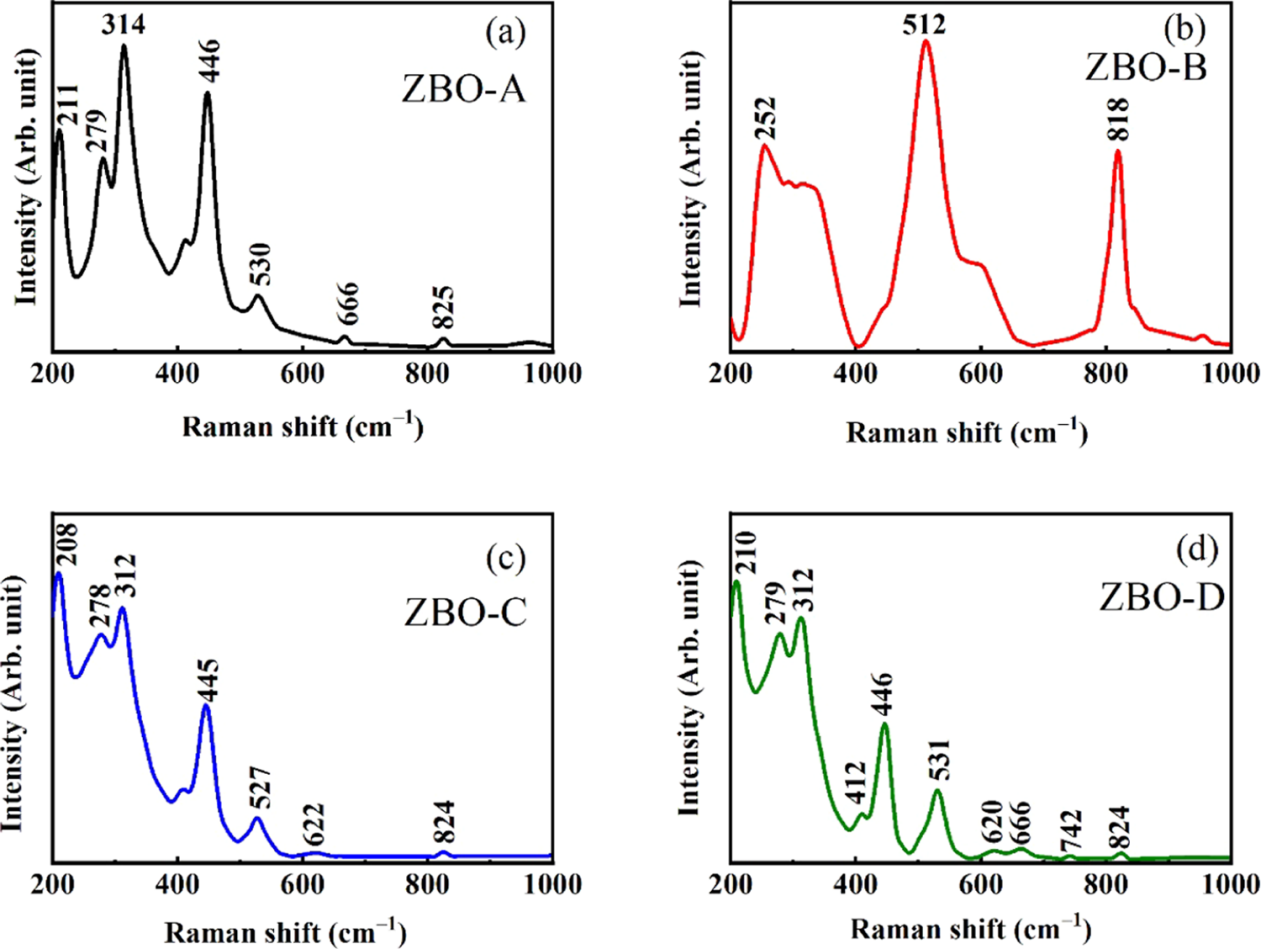
Raman spectrum of (a) ZBO-A, (b) ZBO-B, (c) ZBO-C, and (d) ZBO–D samples.

Raman analysis provides information about the crystal structure, defects, and composition of nanomaterials, semiconductors, polymers, etc.^34^ The Raman spectra of pure and Zn^2+^-doped α-Bi_2_O_3_ nanoparticles in the range 200–1000 cm^–1^ are shown in Figure 5a–d. For the α-Bi_2_O_3_ group, theory states that the optical modes with good agreement of 15A_g_ + 15B_g_ are given by the following equation.^35^
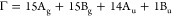
4

Doubly degenerated E_g_ and nondegenerated A_1g_, B_1g_, and B_2g_ modes are first-order active Raman modes. B_1u_ and A_2g_ are the silent modes. Two E_u_ and one A_2u_ modes are associated with longitudinal (LO), transverse optical (TO), and acoustic modes. The Bi–O and Bi–O–Bi vibrations are the origin of bands produced in the region 200 to 550 cm^–1^. The peak around 210 cm^–1^ is due to the vibration of oxygen present in the Bi_2_O_3_ structure. The Bi–O stretching vibrations are associated with A_g_ and B_g_ observed at 314 cm^–1^. Force constants improved with Zn doping; due to this reason, the observed peaks of α-Bi_2_O_3_ nanoparticles shifted toward a higher frequency. The peak observed at 530 cm^–1^ is associated with oxygen vacancies. Raman spectra revealed the Raman active phonon at 622 cm^–1^. A deep investigation of the Raman spectra for ZBO-C and ZBO–D revealed that small intense bands occurred at 666, 724, and 826 cm^–1^, as shown in Figure 5c,d, and these bands represent the rearrangement of the anionic sublattice.^36,37^ The obtained overtones and energy position (cm^–1^) from Raman analysis for pure and Zn^2+^-doped α-Bi_2_O_3_ nanoparticles are shown in Table 2.

**Table 2:** Overtones and Energy Position (cm^–1^) of ZBO-A, ZBO-B, ZBO-C, and ZBO–D Samples

ZBO-A (cm^–1^)	ZBO-B (cm^–1^)	ZBO-C (cm^–1^)	ZBO–D (cm^–1^)	combinations and overtones
211		208	210	B_u_
279	252	278	279	B_g_
314		312	312	(A_g_)
446		445	446	B_g_
530	512	527	531	A_g_
666		622	620	T_2g_
			724	

### 3.4 PL Analysis

**Figure 6:**
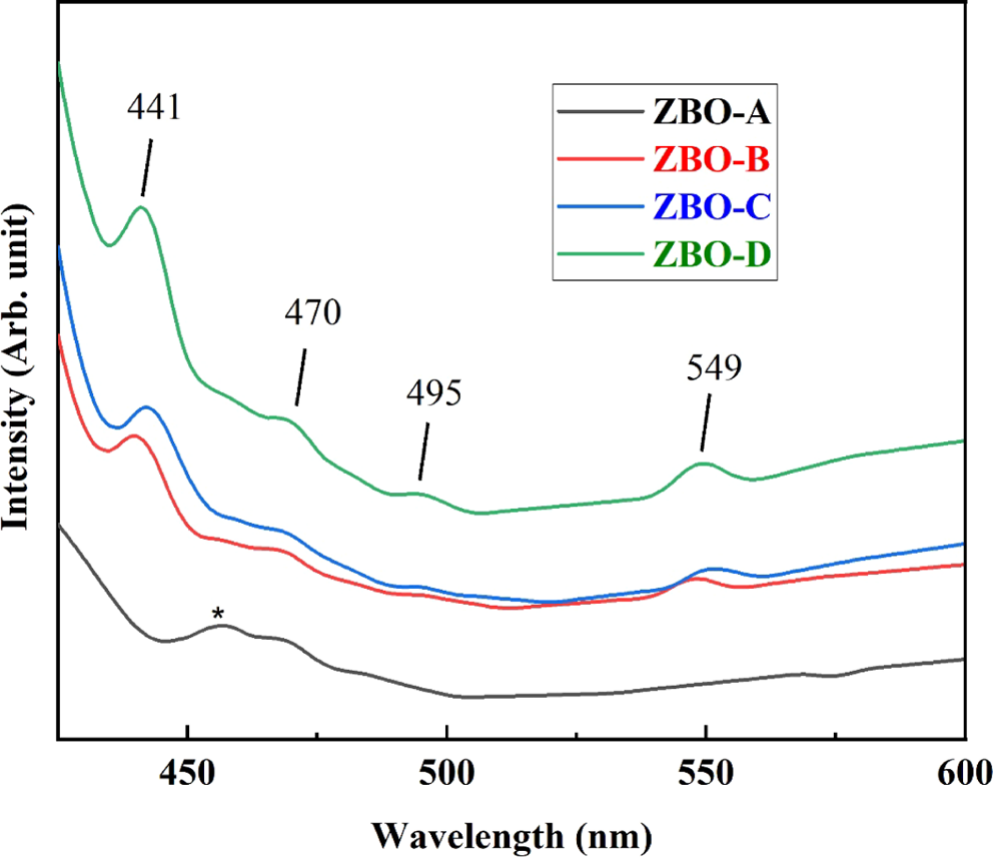
Photoluminescence spectra of pure and Zn^2+^-doped α-Bi_2_O_3_ nanoparticles.

PL emission analysis was recorded at room temperature with 400 nm excitation wavelength for pure and Zn^2+^-doped α-Bi_2_O_3_ nanoparticles. As shown in Figure 6, the major visible emission peak for pure α-Bi_2_O_3_ nanoparticles is observed at 456 nm at room temperature. The valence band of Bi_2_O_3_ is associated with the 6s and 2p orbitals of Bi and O_2_, respectively. Under the irradiation excitation, charge transfers from O_2_ 2p and Bi 6s orbitals to the conduction band of Bi, which is a 6p orbital, and the peak at 465 nm forms due to the recombination of free excitons after the de-excitation.^30,38^ The band edge emission peak at 456 nm for pure α-Bi_2_O_3_ nanoparticles (denoted as * in Figure 6) shifted toward shorter wavelengths with increasing concentration of Zn^2+^ ion in α-Bi_2_O_3_ nanoparticles; e.g., for ZBO–D it occurs at 441 nm. The shifting of band edge emission peak toward the lower wavelength with increasing concentration of Zn^2+^ is due to the improvement in energy of the band-to-band recombination according to the Burstein–Moss effect.^39^ In Zn^2+^-doped α-Bi_2_O_3_ nanoparticles, the other three peaks were also recorded. The first one was recorded at 470 nm, which is produced due to the recombination of Zn interstitial or vacancies with valence band.^40,41^ The second extra peak occurs at 495 nm, which represents the deep-level emission due to surface defects.^42^ The peak located at 549 nm corresponds to the defect emission due to oxygen interstitials or ion vacancies in the structure. This peak is also associated with Zn vacancy in case of zinc oxides.^43,44^ Usually the peak appearing at 549 nm is broad and intense due to oxygen chemisorptions, but in our work, the intensity of the peak is low, which indicates the moderate percenatge.

### 3.5 UV–Vis Spectroscopy

**Figure 7:**
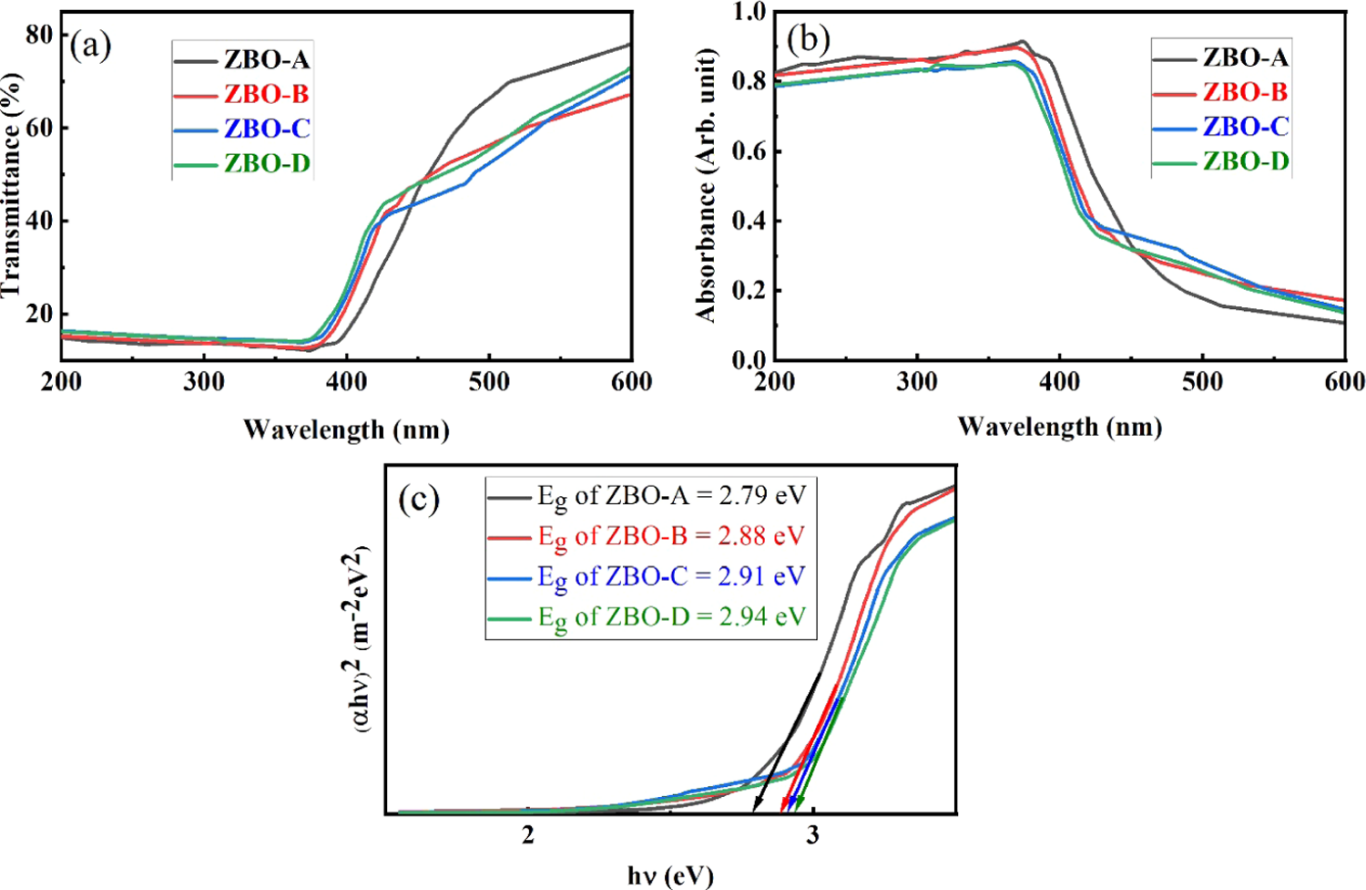
(a) Optical transmittance, (b) optical absorbance, and (c) optical band gap of pure and Zn^2+^-doped α-Bi_2_O_3_ nanoparticles.

The optical transmittance and absorbance of pure and Zn^2+^-doped α-Bi_2_O_3_ nanoparticles in the wavelength range 200 to 600 nm are shown in Figure 7a,b. Figure 7a depicts the optical transmittance of pure and Zn-doped α-Bi_2_O_3_ nanoparticles. The synthesized nanoparticles showed less optical transmittance in the UV region, but in the visible region, nanoparticles exhibited significant optical transmittance. Pure α-Bi_2_O_3_ nanoparticles showed the maximum optical transmittance in the visible region as compared to Zn^2+^-doped α-Bi_2_O_3_ nanoparticles. From Figure 7b, it is clear that the synthesized nanoparticles absorb both ultraviolet and visible regions. The absorption edge of α-Bi_2_O_3_ nanoparticles shifted toward shorter wavelengths with increasing concentration of Zn^2+^ dopant. Absorption analysis indicates that the doping of Zn^2+^ ions introduced the new absorption energy levels because the width of the energy gap progressively improved with the increasing concentration of Zn^2+.^^45,46^ Tauc’s relationship was used for the measurement of optical band gap “*E*_g_” by extrapolating the linear portion of the (α*h*ν)^2^ versus *h*ν (eV) curve.^47^
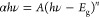
5where the value of “*n*” is 2 for the direct allowed band gap, *h*ν (eV) is the energy of photons, “*A*” is related to the slope of the Tauc line and is the band tailing parameter, and “α” is the absorption coefficient. An increase in optical band gap of the synthesized samples was observed with increasing concentration of Zn^2+^ ions; it increased from 2.79 eV for ZBO-A (pure α-Bi_2_O_3_) to 2.94 eV for ZBO–D. The increase in optical band gap with Zn^2+^ doping is due to Burstein–Moss effect and this shift is already confirmed in the PL analysis section.

### 3.6 SEM and TEM Analysis

**Figure 8:**
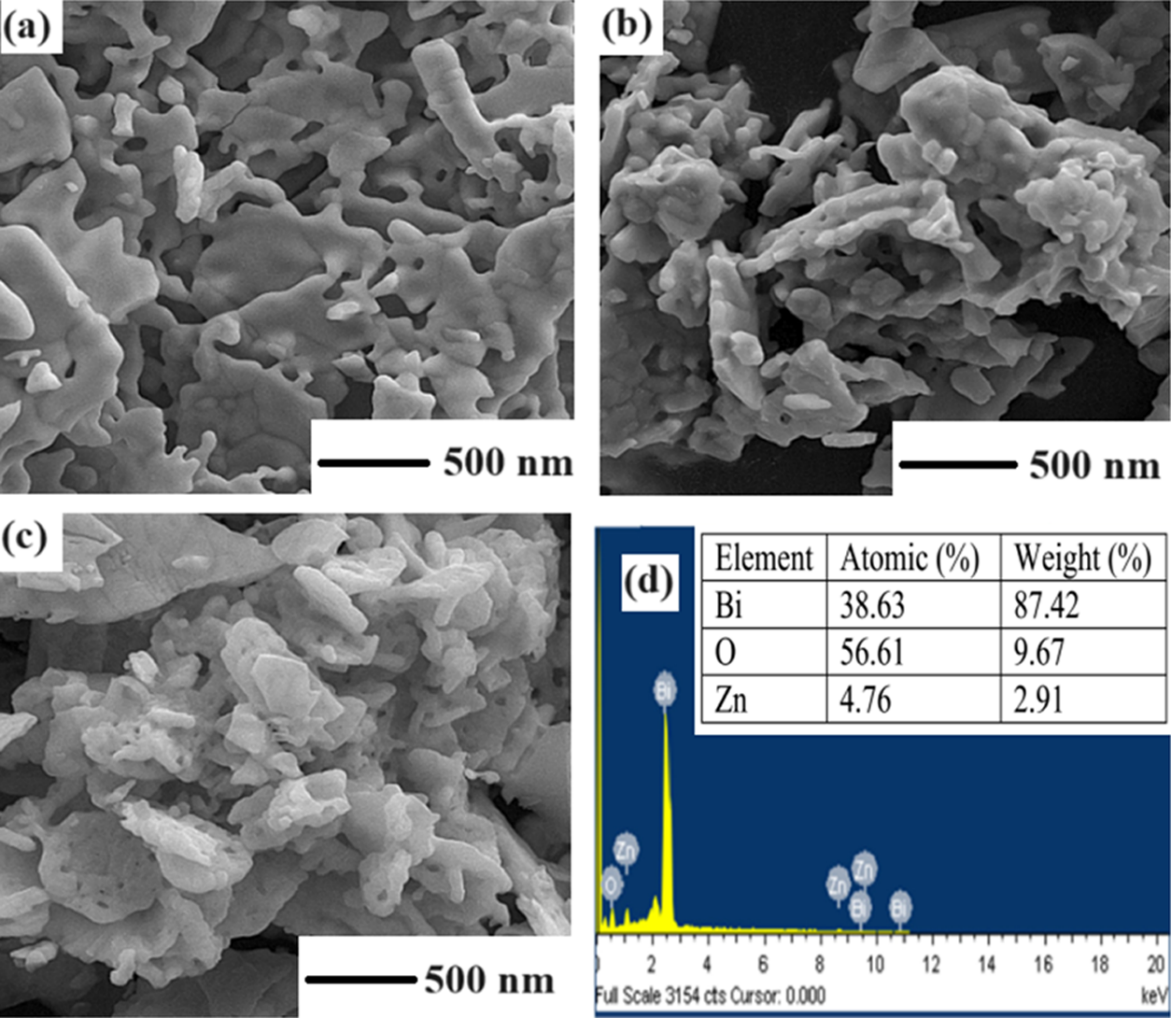
SEM images of (a) ZBO-A, (b) ZBO-B, (c) ZBO-C, and (d) EDS spectra of ZBO-C.

The morphology and elemental composition of the synthesized samples were analyzed by SEM. The SEM images of pure α-Bi_2_O_3_ nanoparticles (ZBO-A) and Zn^2+^-doped α-Bi_2_O_3_ nanoparticles (ZBO-B and ZBO-C) are shown in Figure 8a and 8c,d, respectively. The SEM images show that the fabricated nanoparticles have plate-like morphology. Plate-like morphology provides significantly improved interactions due to a greater surface area. Measurement of particle size is difficult from the obtained SEM images because there is hazy morphology with different particle sizes. SEM also provided the energy dispersive spectroscopy (EDS) spectra of 3% (volume percentage) Zn^2+^-doped α-Bi_2_O_3_ nanoparticles, which is shown in Figure 8d. EDS spectra showed the elemental peaks of Bi and O with addition of Zn peaks.^48^ The EDS results illustrated that the nanoparticles are produced of only the desired atoms without any impurity.

**Figure 9:**
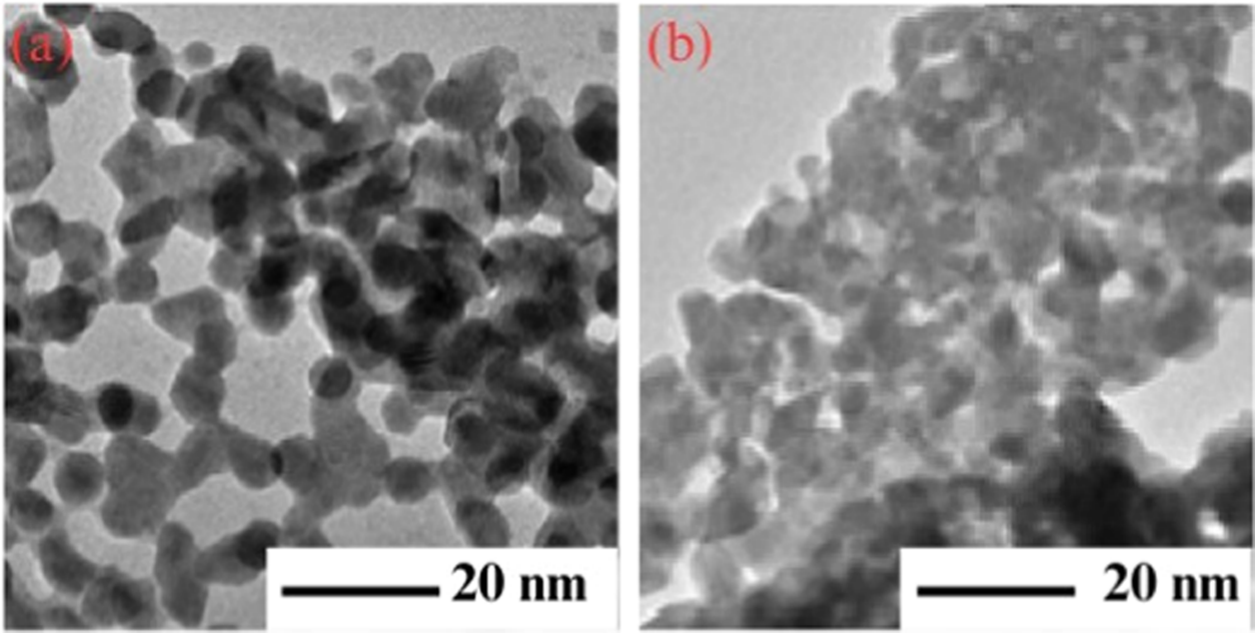
TEM patterns of (a) pure (ZBO-A) and (b) 4.5% Zn^2+^-doped α-Bi_2_O_3_ (ZBO-C) nanoparticles.

For further investigations of the crystalline size and morphology of nanoparticles, TEM analysis was done. Figure 9 shows the TEM images of ZBO-A and ZBO-C samples. Figure 9a illustrates that the pure α-Bi_2_O_3_ nanoparticles are spherical and well separated with sizes of 10–20 nm, while if we compare doped nanoparticles (ZBO-C) with pure ones, the shape of the particles are found to remain spherical but doped nanoparticles are aggregated with sizes of 40–51 nm (Figure 9b).

### 3.7 Electrical Analysis

**Figure 10:**
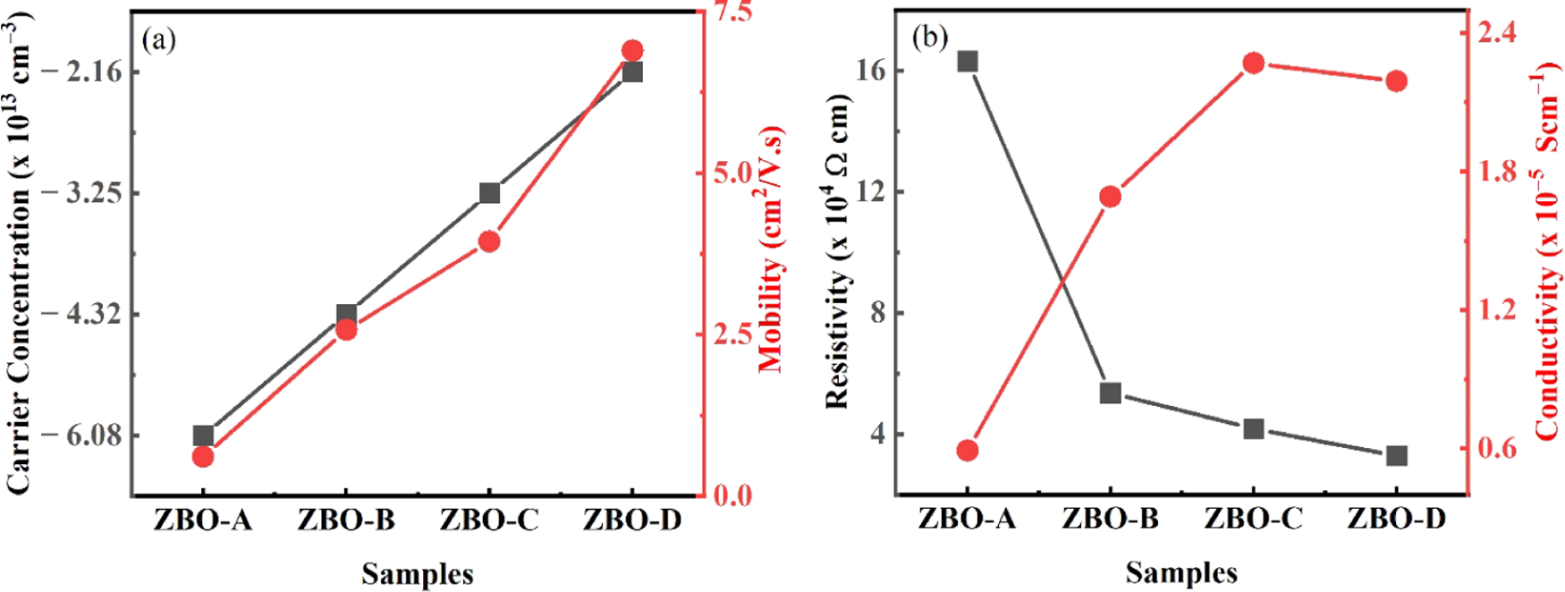
(a) Carrier concentration and mobility. (b) Resistivity and conductivity of pure and doped samples.

To investigate the effect of Zn^2+^ ion on the electrical properties of α-Bi_2_O_3_ nanoparticles, commonly used Hall measurements (Van der Pauw technique) were carried out to measure the carrier concentration, carrier type, resistivity, conductivity, and mobility of the fabricated nanoparticles.^3^ All of the synthesized nanoparticles showed n-type conductivity, and it was also observed that the conductivity and carrier mobility increase from 5.91 × 10^–6^ S cm^–1^ for ZBO-A to 2.19 × 10^–5^ S cm^–1^ for ZBO–D and 0.611 cm^2^/V·s for ZBO-A to 6.89 cm^2^/V·s for ZBO–D, respectively. Maximum conductivity, minimum resistivity, and improved carrier mobility were obtained for ZBO–D. The carrier mobility improved with increase in the volume concentration of Zn^2+^ ions because of the reduction in scattering probability of charge carriers.^49^ The details of carrier concentration, resistivity, conductivity, and mobility are shown in Figure 10 for all samples.

### 3.8 Photocatalytic Analysis

**Figure 11:**
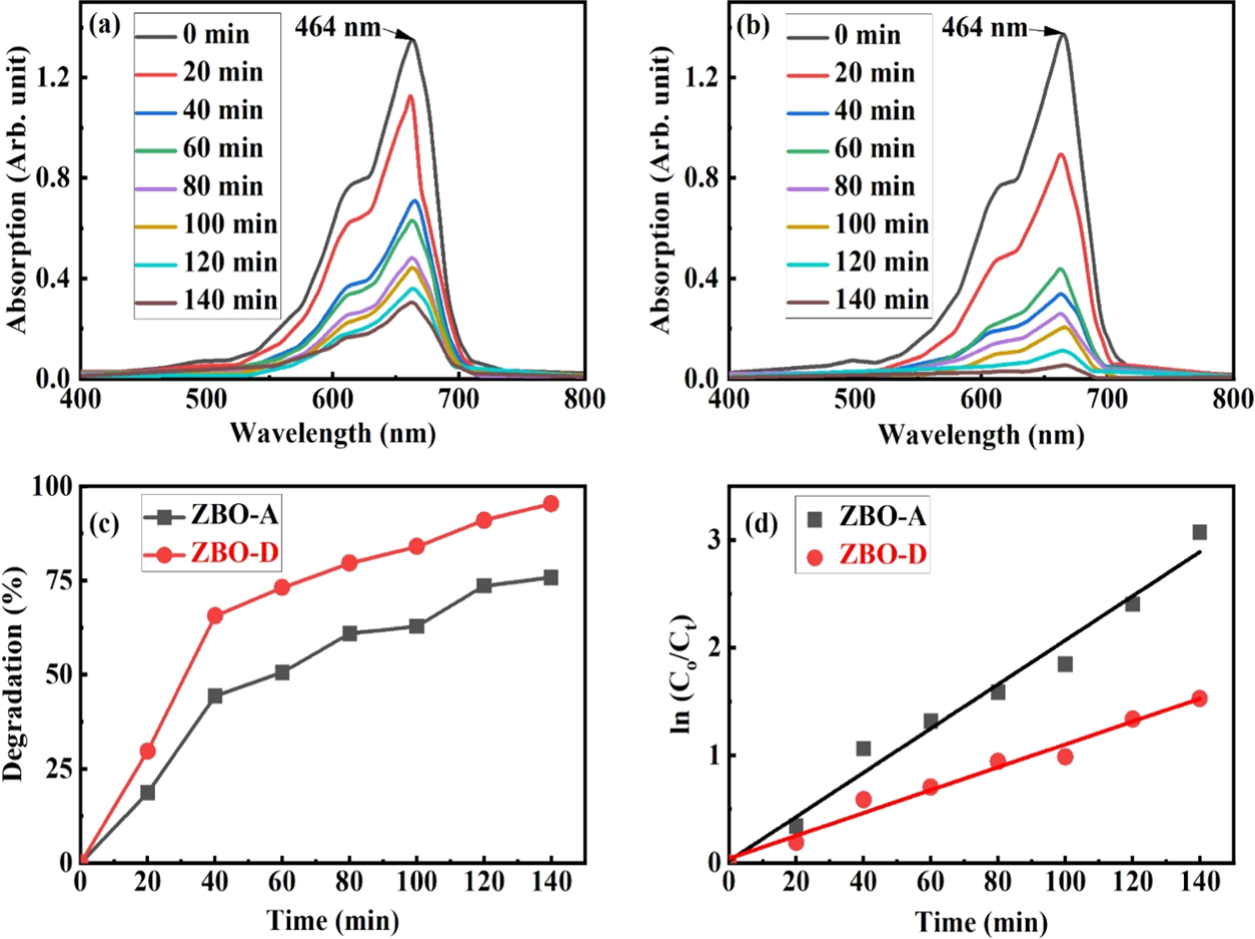
Optical absorption spectrum of MB degradation: (a) pure α-Bi_2_O_3_, (b) Zn^2+^-doped α-Bi_2_O_3_, (c) percentage degradation of organic dye (MB), and (d) linear relation between the logarithm of the relative concentration of MB solution with 0.018 rate constant value and 0.974 linear regression for pure α-Bi_2_O_3_ (ZBO-A) and 0.021 rate constant value and 0.977 linear regression for Zn^2+^-doped α-Bi_2_O_3_ (ZBO–D).

The photocatalytic investigations of pure and Zn^2+^-doped α-Bi_2_O_3_ nanoparticles were carried out on the basis of the percentage degradation of organic dye (MB) in solution under solar irradiation. The change in optical absorbance at 464 nm was measured to calculate the percentage degradation of MB as a function of irradiation interval (20 min). Figure 11a,b shows the variation in optical absorbance as a function of irradiation interval due to the change in concentration of MB in pure (ZBO-A) and Zn^2+^-doped α-Bi_2_O_3_ (ZBO–D). The investigations revealed that pure α-Bi_2_O_3_ exhibits partial degradation and Zn^2+^-doped α-Bi_2_O_3_ shows complete degradation. The results of percentage degradation are shown in Figure 11c, and the doped sample shows a much higher (≈95%) degradation compared with that of the pure sample. A linear relation between the logarithm of the relative concentration (*C*_o_/*C_t_*) of the MB solution and the irradiation time intervals is shown in Figure 11d to monitor the efficiency of degradation. The rate constant values were monitored at 0.018 min^–1^ for pure α-Bi_2_O_3_ and at 0.039 min^–1^ for doped α-Bi_2_O_3_. Doping of Zn improved the photocatalytic activity of α-Bi_2_O_3_ due to electron scavenging.^50−52^

**Figure 12:**
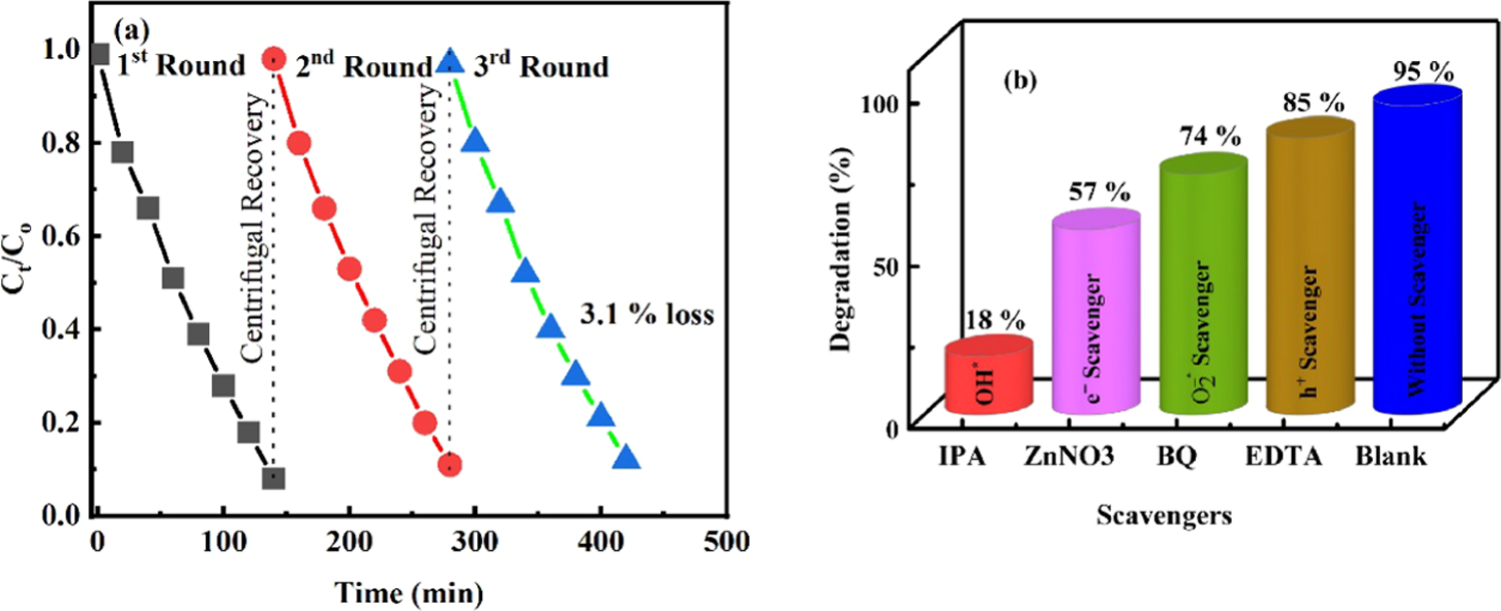
(a) Photostability and (b) scavenging tests of ZBO–D nanocatalyst.

Three recycling studies were carried out on the ZBO–D nanocatalyst to investigate its photostability. At the end of each photocatalytic round, the nanocatalyst was recovered and cleaned for the next round. Figure 12a illustrates the significant photostability of the ZBO–D nanocatalyst during the photocatalytic recycling investigations. At the end of the three recycling investigations, the examined nanocatalyst showed a 3.1% reduction in photocatalytic activity, which is a mere one.

Scavenger experiments were performed to investigate the role of active species in the degradation of MB dye under solar irradiation. Ethylenediaminetetraacetic acid (EDTA), benzoquinone (BQ), ZnNO_3_, and 2-propanol (IPA) were used to remove holes, superoxide radical ions, electrons, and hydroxyl radicals, respectively. Figure 12b illustrated that the ZBO–D nanocatalyst mineralizes approximately 95% of the MB dye after 140 min without any scavenger (mentioned as without a scavenger in Figure 10b). When EDTA was used as the scavenger, the mineralization of MB dye was effective up to 85%. When BQ and ZnNO_3_ were used as scavengers, the mineralization of MB dye was effective up to 74 and 57%, in that order. The dye mineralization efficiency of the ZBO–D nanocatalyst reduced a lot when IPA was used as the scavenger (up to 18%), which illustrates that hydroxyl radicals are the active species in the process of photodegradation.

Photocatalytic activity was improved due to the phenomenon of electron trapping and detrapping, as well as the separation of photogenerated charge carriers. In semiconductor materials, a photoenergy is required to excite the electrons from the valence (*E*_VB_) to conduction band (*E*_CB_). *E*_VB_ and *E*_CB_, the potentials for the ZBO–D nanocatalyst, were measured using the following equations.^53,54^
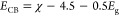
6
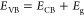
7where χ is the absolute electronegativity and the calculated value of χ for the ZBO–D semiconductor is 6.2. In eq 6, 4.5 represents the free electron energy at the hydrogen scale. The conduction and valence bands for ZBO–D semiconductor are 0.23 and 3.17 eV, respectively.

**Figure 13:**
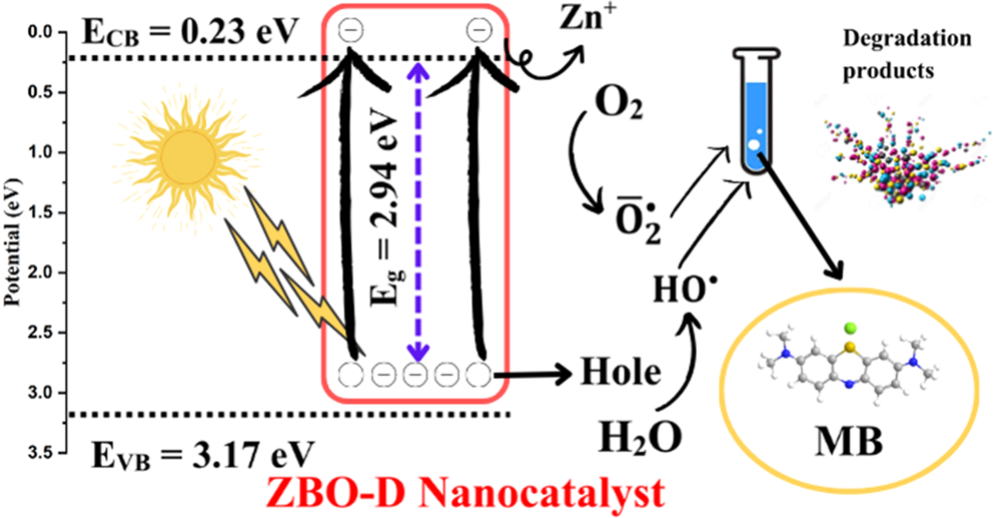
Working mechanism of the ZBO–D nanocatalyst.

The possible reactions in our work are mentioned below. Figure 13 shows the possible mechanism of photocatalytic degradation of the MB dye when ZBO–D is used as a nanocatalyst.
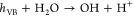

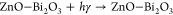

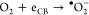

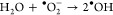

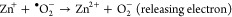

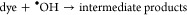

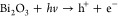

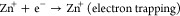

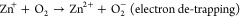


## 4 Conclusions

The citrate sol gel method was used for the synthesis of pure and Zn^2+^-doped α-Bi_2_O_3_ nanoparticles with varying volume concentrations of zinc. XRD analysis show that all of the synthesized nanoparticles were formed in monoclinic stable phase (α-phase). Optical analysis revealed that the introduction of Zn^2+^ ions into the bismuth oxide lattice generated the new energy levels and improved the optical band gap. Doping of Zn^2+^ ions improved the conductivity and carrier charge mobility of the α-Bi_2_O_3_ nanostructures. The tuned optical band gap, improved conductivity and carrier mobility, and significantly reduced resistivity make these Zn^2+^-doped α-Bi_2_O_3_ nanoparticles a better candidate for optoelectronic devices such as photovoltaic applications. The optimum doping level of zinc (4.5% Zn^2+^-doped α-Bi_2_O_3_) reveals the attractive photocatalytic activity of α-Bi_2_O_3_ nanostructures due to electron trapping and detrapping for solar cells.
